# Prenatal Polybrominated Diphenyl Ether Exposures and Neurodevelopment in U.S. Children through 5 Years of Age: The HOME Study

**DOI:** 10.1289/ehp.1307562

**Published:** 2014-05-28

**Authors:** Aimin Chen, Kimberly Yolton, Stephen A. Rauch, Glenys M. Webster, Richard Hornung, Andreas Sjödin, Kim N. Dietrich, Bruce P. Lanphear

**Affiliations:** 1 Division of Epidemiology and Biostatistics, Department of Environmental Health, University of Cincinnati College of Medicine, Cincinnati, Ohio, USA; 2 Division of General and Community Pediatrics, Department of Pediatrics, Cincinnati Children’s Hospital Medical Center, Cincinnati, Ohio, USA; 3 Child and Family Research Institute, BC Children’s and Women’s Hospital and Faculty of Health Sciences, Simon Fraser University, Vancouver, British Columbia, Canada; 4 Division of Laboratory Sciences, National Center for Environmental Health, Centers for Disease Control and Prevention, Atlanta, Georgia, USA

## Abstract

Background: Polybrominated diphenyl ethers (PBDEs) are persistent chemicals that have been widely used as flame retardants in furniture, carpet padding, car seats, and other consumer products during the past three decades.

Objective: We examined whether *in utero* exposure to PBDEs is associated with child cognitive function and behavior in a U.S. study sample.

Methods: In a prospective birth cohort, we measured maternal serum concentrations of BDE-47 and other PBDE congeners in 309 women at 16 weeks of gestation during 2003–2006 and followed their children in Cincinnati, Ohio. We measured cognitive and motor abilities using the Bayley Scales of Infant Development-II at ages 1, 2, and 3 years; intelligence using the Wechsler Preschool and Primary Scale of Intelligence-III at age 5 years; and children’s behaviors using the Behavioral Assessment System for Children-2 annually at ages 2–5 years. We used linear mixed models or generalized estimating equations with adjustment for potential confounders to estimate associations between these outcomes and log_10_-transformed PBDE concentrations.

Results: The geometric mean of BDE-47 in maternal serum (20.1 ng/g lipid) was comparable with U.S. adult national reference values. Prenatal BDE-47 was not significantly associated with Bayley Mental or Psychomotor Development Indices at 1–3 years, but a 10-fold increase in prenatal BDE-47 was associated with a 4.5-point decrease (95% CI: –8.8, –0.1) in Full-Scale IQ and a 3.3-point increase (95% CI: 0.3, 6.3) in the hyperactivity score at age 5 years.

Conclusions: Prenatal exposure to PBDEs was associated with lower IQ and higher hyperactivity scores in children.

Citation: Chen A, Yolton K, Rauch SA, Webster GM, Hornung R, Sjödin A, Dietrich KN, Lanphear BP. 2014. Prenatal polybrominated diphenyl ether exposures and neurodevelopment in U.S. children through 5 years of age: the HOME study. Environ Health Perspect 122:856–862; http://dx.doi.org/10.1289/ehp.1307562

## Introduction

Polybrominated diphenyl ethers (PBDEs) are flame retardants added to furniture, carpet padding, electronic devices, and other consumer products. Since their introduction in the 1970s, humans have accumulated PBDEs in their tissues via dust ingestion, dietary intake, absorption from dermal contact, and inhalation [Centers for Disease Control and Prevention (CDC) 2009; Lorber 2008]. The body burden of PBDEs in Americans is about 10 times higher than in Europeans or Asians because of previous U.S. regulations (California Technical Bulletin 117) that required the addition of flame retardants to prevent burn injuries and property damage (Sjödin et al. 2008; Stapleton et al. 2012). The PBDEs have 209 lipophilic congeners; the major congeners (e.g., BDEs 47, 99, 100, 153) are mostly found in the pentaBDE commercial mixture used in polyurethane foams, and they have long half-lives (2–7 years) in humans (Geyer et al. 2004). Concerns have been raised about potential toxicity of PBDEs, focused mainly on thyroid-disrupting effects and developmental neurotoxicity (Costa et al. 2008). In particular, hyperactivity, decreased habituation, and deficits in learning and memory have been reported in experimental animal studies (Costa and Giordano 2007).

Serum PBDE concentrations have been associated with reduced IQ and increased scores of attention problems, hyperactivity, and attention deficit/hyperactivity disorder (ADHD) index in children. A prenatal cohort established following the 11 September 2001 World Trade Center (WTC) attack in New York City reported a decrease in Full-Scale IQ (FSIQ) associated with increased levels of BDEs 47, 99, 100, or 153 congeners in cord serum (Herbstman et al. 2010). A 10-fold increase in cord serum BDE-47 was associated with a 5.5-point decrease (95% CI: –10.8, –0.2) in FSIQ at 4 years of age (Herbstman et al. 2010). In a recent study involving children of migrant farm workers, prenatal exposure to PBDEs was associated with lower FSIQ up to 7 years of age and higher ADHD index (Eskenazi et al. 2013). Collectively, these studies suggest that prenatal PBDE exposures are associated with deficits in intellectual abilities and increased behavior problems in children. We hypothesized that prenatal PBDE exposure would be associated with decrements in cognitive abilities and increases in hyperactivity behaviors in 5-year-old children enrolled in a U.S. birth cohort.

## Methods

*Study participants*. Pregnant women were enrolled in the Health Outcomes and Measures of the Environment (HOME) Study between March 2003 and February 2006 in Cincinnati, Ohio. The study was designed to investigate neurobehavioral and health effects of exposures to low-level, environmental toxicants or suspected toxicants. Eligibility criteria for HOME study mothers were *a*) being pregnant at 16 (± 3) weeks of gestation; *b*) being ≥ 18 years old; *c*) residing in a house built before 1978 (which may contain leaded paint, a focus of another part of the overall study); *d*) having no history of human immunodeficiency virus (HIV) infection; and *e*) not taking medication for seizures or thyroid disorders. A total of 389 pregnant women participated and delivered live singleton infants. The children were followed up at 1, 2, 3, 4, and 5 years of age for assessment of neurodevelopment, physical growth, and health conditions. We retained 309 mothers and their children in this analysis after excluding infants with major birth defects (*n* = 2), mothers with insufficient serum for PBDE measurement (*n* = 30), and those who did not complete any neurobehavioral assessments between 1 and 5 years of age (*n* = 48). The HOME study was approved by the institutional review boards at the Cincinnati Children’s Hospital Medical Center (CCHMC) and the CDC. The study mothers gave informed consent before enrollment in the study and at postnatal follow-up visits for their children’s participation.

*Maternal serum PBDE measurement*. We measured serum PBDE concentrations during early pregnancy. The women provided blood samples at the prenatal enrollment visit around 16 weeks of gestation. The serum samples were stored at –80°C and shipped to the CDC for testing of environmental contaminants. The PBDE congeners (BDEs 17, 28, 47, 66, 85, 99, 100, 153, 154, 183) were measured at the CDC’s Persistent Organic Pollutants Biomonitoring Laboratory at the National Center for Environmental Health (NCEH), using gas chromatography/isotope dilution high-resolution mass spectrometry (Jones et al. 2012). The serum samples were pretreated and extracted by solid phase extraction. Blank samples were included in a set of test samples and quality control samples. The serum PBDE concentrations were calculated on a lipid basis (nanograms per gram lipid, including total cholesterol and triglycerides) to account for their lipophilic property. All 309 subjects (100%) had detectable BDE-47. However, 30 subjects did not have enough serum for reliable quantification of other PBDE congeners, leaving 279 subjects tested for BDEs 99, 100, and 153. Among the 279 subjects, 6 had concentrations of BDEs 99, 100, or 153 below the limit of detection (LOD) (Sjödin et al. 2008), and their values were replaced with the LOD divided by the square root of 2 (Hornung and Reed 1990). To maximize power and representativeness of total PBDE exposure, we focused on serum BDE-47, which accounted for 51% of all serum PBDE congeners and was available for all 309 subjects. In secondary analyses, we examined the association of the sum of four PBDE congeners (sum_4_: BDEs 47, 99, 100, 153, accounting for 90% of the sum of all 10 congeners tested) in 279 subjects.

*Developmental and behavioral assessments*. We administered the Bayley Scales of Infant Development-II (BSID-II) (Bayley 1993) to evaluate cognitive and motor development during follow-up visits of the children at 1, 2, and 3 years of age. The BSID-II yields Mental Development Index (MDI) and Psychomotor Development Index (PDI) standard scores. When the children were 5 years of age, we administered the Wechsler Preschool and Primary Scale of Intelligence-III (WPPSI-III) to obtain FSIQ (Wechsler 2004). The MDI and FSIQ both measure cognitive function and have a population mean of 100 and a standard deviation of 15. When children were 2, 3, 4, and 5 years, their parents completed the Behavioral Assessment System for Children-2 (BASC-2), which offers a comprehensive assessment of a child’s adaptive and problem behaviors in community and home settings (Reynolds and Kamphaus 2004). It provides four composite scores for Externalizing Problems, Internalizing Problems, Behavioral Symptoms Index, and Adaptive Skills, as well as 12 clinical subscales and 5 content scales. Externalizing Problems include subscales of aggression and hyperactivity; Internalizing Problems include subscales of anxiety, depression, and somatization; Behavioral Symptoms Index includes subscales of aggression, hyperactivity, depression, atypicality, withdrawal, and attention problems; Adaptive Skills include subscales of activity of daily living, adaptability, social skills, and functional communication. The hyperactivity subscale is included in both Externalizing Problems and Behavioral Symptoms Index composite scores. The BASC-2 scores have a mean of 50 and a standard deviation of 10. Except for Adaptive Skills and the corresponding subscales, higher BASC-2 scores suggest nonoptimal behavior.

All clinical assessments were performed by HOME Study staff trained and certified by a developmental psychologist (K.Y.). The assessors conducted the neurobehavioral assessments without knowledge of maternal PBDE levels.

*Statistical analyses*. We focused our statistical analyses on the associations between prenatal PBDE exposures and intellectual abilities and externalizing behavior problems and hyperactivity because of animal study evidence of hyperactivity and deficits in learning and memory (Costa and Giordano 2007). In secondary analyses, we also examined Bayley PDI, BASC-2 Internalizing Problems, Behavioral Symptoms Index, Adaptive Skills, and BASC-2 subscales for attention problems, aggression, anxiety, and withdrawal. The PBDE concentrations were log_10_ transformed because of the right-skewed distribution. We first estimated adjusted associations between log_10_ transformed maternal BDE-47 and FSIQ and externalizing behaviors at 5 years of age using generalized additive models (GAM) to examine whether the dose–response patterns were approximately linear. We used linear mixed models to estimate regression estimates of PBDEs to take advantage of the longitudinal design and repeated measurements of cognition and behavior in children. We examined associations between maternal PBDEs and neurobehavioral outcomes at different ages by entering interaction terms between PBDEs (continuous variable) and child age (categorical variable) in the mixed models. We constructed a linear mixed model for MDI at 1, 2, and 3 years and FSIQ at 5 years because MDI and FSIQ are statistically equivalent, and MDI at 3 years and FSIQ at 5 years were highly correlated in this study, with Pearson correlation coefficient = 0.72. We constructed a linear mixed model for Externalizing Problems at 2, 3, 4, and 5 years. We also constructed another linear mixed model for hyperactivity subscale at 2, 3, 4, and 5 years. In secondary analyses, we established a linear mixed model for PDIs at 1, 2, and 3 years. Additionally, we used separate linear mixed models for Internalizing Problems, Behavior Symptoms Index, Adaptive Skills, and BASC-2 subscales for attention problems, aggression, anxiety, and withdrawal at 2, 3, 4, and 5 years. We used an unstructured covariance structure in the linear mixed models based on the Akaike information criterion after comparing with an autoregressive covariance structure.

For all multiple regression models, we included *a priori* the following potential covariates: maternal age at enrollment, maternal race/ethnicity, education, marital status, maternal serum cotinine concentrations at enrollment, maternal IQ [assessed by Wechsler Abbreviated Scale of Intelligence (WASI) (Wechsler 1999)], and child sex (Eskenazi et al. 2013; Herbstman et al. 2010). We also included maternal depression [Beck Depression Inventory II score at enrollment (Beck et al. 1996)], household income, and Home Observation for Measurement of the Environment (HOME) Inventory (Caldwell and Bradley 1984) measured at a 1-year home visit as covariates for adjustment in the multiple regression models because of their correlations with both prenatal PBDE concentrations and FSIQ at 5 years of age (*p* < 0.05). The potential covariates were coded as categorized or continuous variables as shown in Tables 1 and 2. Finally, we examined maternal blood lead concentration (mean, 0.7 μg/dL) as a potential confounder, but it did not correlate with PBDEs (*r* = 0.09, *p* > 0.05), nor did it modify the regression coefficients of PBDEs on MDI/FSIQ, Externalizing Problems, and hyperactivity subscale by > 10%, and thus was not included in the final models.

**Table 1:** Maternal serum PBDE concentrations around 16 weeks of gestation and child neurodevelopmental test scores at age 5 years by maternal and child characteristics.

Categorical characteristics	*n* at enrollment (%)	Maternal BDE-47 (ng/g lipid) [GM (GSD)]	*n* with WPPSI at age 5 (%)	WPPSI FSIQ at age 5 (mean ± SD)	*n* with BASC-2 at age 5 (%)	BASC externalizing problems at age 5 (mean ± SD)
All participants	309 (100)	20.1 (2.61)	190 (100)	102.33 ± 15.20	194 (100)	47.13 ± 9.26
Maternal age (years)*^,^**
< 25	63 (20)	27.54 (2.24)	39 (21)	92.18 ± 13.60	39 (20)	48.71 ± 13.11
25–34	194 (63)	19.50 (2.69)	122 (64)	103.59 ± 14.47	128 (66)	46.78 ± 7.86
≥ 35	52 (17)	15.14 (2.57)	29 (15)	110.69 ± 13.49	27 (14)	46.44 ± 8.89
Maternal race/ethnicity*^,^**
Non-Hispanic white	207 (67)	16.98 (2.51)	122 (64)	107.09 ± 13.55	124 (64)	47.48 ± 8.34
Non-Hispanic black and others	102 (33)	28.84 (2.51)	68 (36)	93.79 ± 14.34	70 (36)	46.51 ± 10.74
Maternal education*^,^**
High school or less	70 (23)	28.84 (2.29)	44 (23)	90.23 ± 13.65	45 (23)	47.91 ± 11.35
Some college or 2-year degree	72 (23)	22.91 (2.34)	50 (26)	98.58 ± 13.40	50 (26)	49.52 ± 10.69
Bachelor’s	101 (33)	16.98 (2.69)	60 (32)	110.48 ± 12.14	61 (31)	45.25 ± 6.22
Graduate or professional	66 (21)	15.14 (2.82)	36 (19)	108.75 ± 12.63	38 (20)	46.08 ± 8.06
Maternal marital status*^,^**
Married or living with partner	251 (81)	18.20 (2.63)	149 (78)	105.52 ± 14.26	153 (79)	46.52 ± 8.09
Not married and living alone	58 (19)	31.63 (2.19)	41 (22)	90.73 ± 12.81	41 (21)	49.39 ± 12.60
Household income*^,^**^,#^
< $20,000	63 (20)	32.36 (2.29)	44 (23)	88.45 ± 13.34	45 (23)	51.02 ± 12.56
$20,000–79,999	157 (51)	19.95 (2.63)	96 (51)	103.63 ± 13.63	97 (50)	45.63 ± 8.03
≥ $80,000	89 (29)	14.45 (2.40)	50 (26)	112.06 ± 10.13	52 (27)	46.56 ± 7.01
Child sex*
Male	141 (46)	17.78 (2.51)	83 (44)	100.40 ± 15.35	87 (45)	47.38 ± 8.24
Female	168 (54)	22.39 (2.63)	107 (56)	103.83 ± 15.00	107 (55)	46.93 ± 10.05
GM, geometric mean; GSD, geometric standard deviation. *p < 0.05 for BDE-47. **p < 0.05 for FSIQ. ^#^*p *< 0.05 for BASC Externalizing Problems. The statistical tests included *t*-test, analysis of variance, or Student–Newman–Keuls test.						

**Table 2:** Pearson correlation coefficients of continuous characteristics with maternal serum PBDE concentrations around 16 weeks of gestation, and child neuro­developmental test scores at age 5 years.

Continuous characteristics	*n* at enrollment	Mean ± SD	Pearson *r* with maternal BDE-47(log_10_ ng/g lipid)	*n* with WPPSI at age 5	Mean ± SD	Pearson *r* with WPPSI FSIQ at age 5	*n* withBASC‑2 at age 5	Mean ± SD	Pearson *r* with BASC externalizing problems at age 5
Maternal IQ	301	107.16 ± 14.6	–0.24*	186	105.97 ± 15.4	0.50**	190	106.13 ± 15.5	–0.05
Maternal BDI depression score at baseline	307	9.60 ± 6.82	0.23*	189	9.75 ± 6.80	–0.26**	193	9.70 ± 6.75	0.29^#^
Maternal cotinine at baseline [GM (GSD)] (ng/mL)	309	0.04 (18)	0.29*	190	0.04 (14)	–0.34**	194	0.04 (14)	0.10
HOME score at age 1 visit	297	39.36 ± 5.26	–0.27*	183	39.11 ± 5.55	0.45**	187	39.13 ± 5.61	–0.02
BDI, Beck Depression Index; GM, geometric mean; GSD; geometric standard deviation. *p < 0.05 for BDE-47. **p < 0.05 for FSIQ. ^#^*p *< 0.05 for BASC Externalizing Problems.									

We also examined binary measures for a child to be considered “at risk” of clinically significant developmental deficits (MDI/WPPSI FSIQ < 85, PDI < 85) or behavioral problems (Externalizing Problems score ≥ 60, Internalizing Problems score ≥ 60, Behavioral Symptoms Index ≥ 60, Adaptive Skills score < 40, hyperactivity subscale score ≥ 60). We used adjusted generalized linear models (GLM) with generalized estimating equations (GEE) to estimate associations with repeated measurements from 1 through 5 years.

*Sensitivity analyses*. We tested the interaction of BDE-47 and child sex on neurobehavioral outcomes by including an interaction term of BDE-47 (continuous variable) and sex (dichotomous variable) in the regression models with repeated measurements. We repeated the analyses after excluding 16 mothers with BDE-47 > 100 ng/g lipid to evaluate the potential influence of PBDE concentrations higher than the 95th percentile on regression estimates. We assessed the potential impact of sample selection by examining the difference in demographics and socioeconomic status between the analytical sample of 309 subjects and the 80 subjects not included due to missing exposure or outcome assessments. We further examined the difference in demographics, socioeconomic status, maternal PBDE concentrations, and Bayley scores between subjects with and without follow-up at 5 years of age among the analytical sample of 309 subjects. The statistical computing was completed with SAS version 9.3 (SAS Institute Inc., Cary, NC, USA), and the graphing was produced in R version 2.15 (R Developmental Team; www.r-project.org/). The statistical tests were two-sided with a significance level at a *p*-value of 0.05. For tests for statistical interactions, the significance level was set at a *p*-value of 0.10.

## Results

The maternal serum PBDE concentrations and child neurodevelopmental test scores at 5 years of age are shown by maternal and child characteristics (Table 1). The mean (± SD) age of 309 pregnant women at enrollment was 29.9 ± 5.6 years, 67% were non-Hispanic white, and 54% had a Bachelor’s degree or graduate education. The 309 women with BDE-47 data included in the analysis were more likely to be non-Hispanic white, older, and have higher education and household income than the 80 women from the original HOME study cohort who were excluded due to missing exposure or neurobehavioral assessment (data not shown). However, the 190 child participants who had an IQ test at age 5 years were not statistically different in demographics, socioeconomic status, maternal PBDE concentrations, and Bayley scores at ages 1–3 years from the 119 participants who did not complete IQ test at age 5 years (see Supplemental Material, Table S1).

The geometric mean (GM) of BDE-47 in maternal serum was 20.1 ng/g lipid (median, 18.9 ng/g lipid; 10th–90th percentile, 6.4–67.9 ng/g lipid), and the GM of sum_4_BDEs was 37.7 ng/g lipid (median, 34.6 ng/g lipid; 10th–90th percentile, 12.0–129.2 ng/g lipid). The Pearson correlation coefficient between log_10_-transformed BDE-47 and sum_4_BDEs was 0.94 (*p* < 0.05).

Mothers who were non-Hispanic black and other race/ethnicity, or unmarried and not living with a partner had significantly higher serum BDE-47 concentrations, and their children had lower FSIQ at age 5 years (Table 1). Mothers < 25 years of age had the highest BDE-47 concentrations, and their children had the lowest FSIQ in the three age groups (Table 1). Mothers with household income < $20,000 had the highest BDE-47 concentrations, and their children had the lowest FSIQ and the highest Externalizing Problems score in the three income groups (Table 1). Mothers with high school or less education had higher BDE-47 concentrations than mothers with Bachelor’s or higher degree, and their children had the lowest FSIQ in the four education groups (Table 1). Maternal IQ and HOME Inventory scores were inversely correlated with serum BDE-47 concentration and positively correlated with child FSIQ, whereas high maternal depression score and higher serum cotinine levels were correlated with higher BDE-47 concentration and lower FSIQ (Table 2). Externalizing Problems scores were higher in children of mothers who had a higher depression symptom score (Table 2).

**Figure 1:**
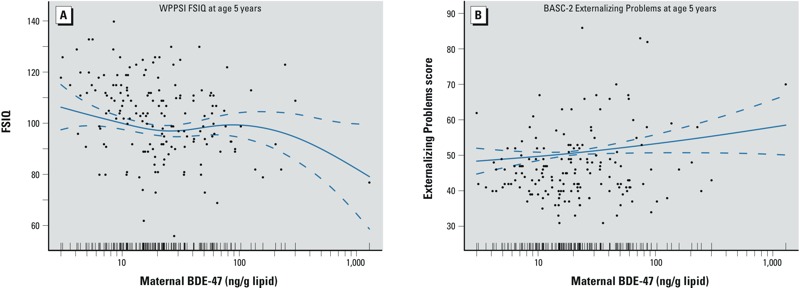
Scatter plots of maternal serum BDE‑47 concentrations and child cognitive and behavior rating scores at age 5 years with generalized additive model curve fitting: (*A*) WPPSI FSIQ; (*B*) BASC-2 Externalizing Problems. Data points represent paired data from each mother–child dyad. Solid lines indicate the natural cubic spline of the covariates adjusted association, and dotted lines represent 95% CIs. The density of BDE‑47 distribution is shown as vertical bars on the log_10_-transformed *x*‑axis. Covariates adjusted include maternal age at enrollment, race, education, marital status, maternal serum cotinine concentrations at enrollment, maternal IQ, child sex, maternal depression, household income, and HOME Inventory score.

**Figure 2:**
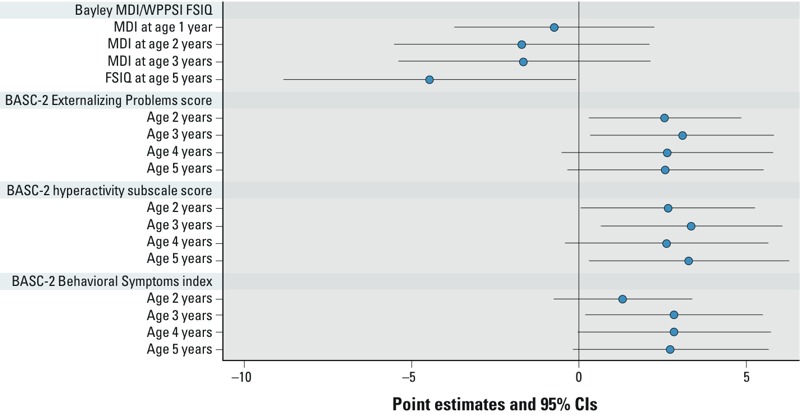
Estimated score differences and 95% CIs in cognitive and behavior rating scores at ages 1–5 years by a 10-fold increase in prenatal PBDE concentrations: Bayley MDI/WPPSI FSIQ, BASC-2 Externalizing Problems score, BASC-2 hyperactivity subscale score, and BASC-2 Behavioral Symptoms Index. The numbers of children in the covariate adjusted mixed model for Bayley MDI/WPPSI FSIQ were 285 at 1 year, 239 at 2 years, 220 at 3 years, and 179 at 5 years of age. The numbers of children in the mixed model for BASC-2 outcomes were 240 at 2 years, 227 at 3 years, 165 at 4 years, and 183 at 5 years of age. Covariates adjusted include maternal age at enrollment, race, education, marital status, maternal serum cotinine concentrations at enrollment, maternal IQ, child sex, maternal depression, household income, and HOME Inventory.

There was an inverse association of maternal BDE-47 concentrations with child FSIQ (Figure 1A). In repeated measurement analyses, a 10-fold increase in maternal BDE-47 exposure was associated with a significantly lower FSIQ score at age 5 years (–4.5 points; 95% CI: –8.8, –0.1) (Figure 2). A 10-fold increase in maternal BDE-47 was associated with lower average MDI scores at 1, 2, and 3 years of age, but differences were small and not statistically significant.

Maternal BDE-47 concentrations were associated with higher Externalizing Problems scores at age 5 years (Figure 1B). In repeated measurement analysis, maternal BDE-47 concentrations were positively associated with Externalizing Problems score at ages 2 and 3 years with statistical significance [e.g., 3.1 points higher at 3 years of age (95% CI: 0.3, 5.8) in association with a log_10_ increase in BDE-47] (Figure 2). At ages 4 and 5 years, the associations were positive but not statistically significant [e.g., 2.6 points higher (95% CI: –0.4, 5.5) at 5 years of age] (Figure 2). Hyperactivity subscale scores in early childhood also were positively and significantly associated with maternal BDE-47 concentrations [e.g., 3.3 points higher (95% CI: 0.3, 6.3) at age 5 years] (Figure 2). Maternal BDE-47 concentration was not significantly associated with the Bayley PDI, BASC-2 composite scores for Adaptive Skills or Internalizing Problems (which includes anxiety, depression, and somatization subscales), or BASC-2 subscales for attention problems, aggression, anxiety, withdrawal behaviors (see Supplemental Material, Table S2). However, maternal BDE-47 was positively associated with Behavioral Symptoms Index (which includes subscales for hyperactivity, aggression, attention problems, and withdrawal, as well as depression and atypicality subscales) (Figure 2). Associations of sum_4_BDEs and MDI/FSIQ and BASC-2 outcomes (Externalizing Problems, Behavioral Symptoms Index, and hyperactivity subscale scores) were very similar to corresponding associations with BDE-47 (see Supplemental Material, Table S3).

MDI/FSIQ < 85 was not significantly associated with maternal PBDE concentrations (Table 3). However, maternal exposure to BDE-47 was associated with higher odds of being in the “at risk” range for Externalizing Problems, hyperactivity subscale score, and Behavioral Symptom Index (Table 3). For outcomes at 5 years of age, the odds ratios (ORs) and 95% CIs for a log_10_ increase in maternal BDE-47 were 2.66 (95% CI: 0.47, 15.06) for Externalizing Problems score ≥ 60 (*n* = 17), 4.45 (95% CI: 1.31, 15.14) for hyperactivity subscale score ≥ 60 (*n* = 19), and 4.80 (95% CI: 1.19, 19.26) for Behavioral Symptoms Index ≥ 60 (*n* = 16). The association between BDE-47 and Adaptive Skills score < 40 was not statistically significant in any of the ages from 2 through 5 years. At 5 years of age, the OR and 95% CI for a log_10_ increase in maternal BDE-47 were 0.80 (95% CI: 0.29, 2.14) for Adaptive Skills score < 40 (*n* = 12). The association between maternal BDE-47 and Internalizing Problems score ≥ 60 was not statistically significant in any of the ages from 2 through 5 years. At 5 years of age, the OR and 95% CI for a log_10_ increase in maternal BDE-47 were 2.37 (95% CI: 0.84, 6.63) for Internalizing Problems score ≥ 60 (*n* = 10). The results of sum_4_BDEs and at risk for clinically significant developmental deficits (MDI/FSIQ < 85, Externalizing Problems score ≥ 60, hyperactivity subscale score ≥ 60, Behavioral Symptoms Index ≥ 60) were similar to corresponding results of BDE-47 (see Supplemental Material, Table S4).

**Table 3:** Estimated ORs (95% CIs) of having cognitive and behavior rating scores 1 SD from the population mean at ages 1–5 years by a 10-fold increase in maternal BDE‑47 concentrations.

Age (years)	MDI/FSIQ < 85		Externalizing Problems ≥ 60		Hyperactivity subscale ≥ 60		Behavioral Symptoms Index ≥ 60	
	*n* (%)	OR (95% CI)	*n* (%)	OR (95% CI)	*n* (%)	OR (95% CI)	*n* (%)	OR (95% CI)
1	52 (18.25)	1.22 (0.51, 2.91)	—	—	—	—	—	—
2	78 (32.64)	0.83 (0.39, 1.78)	12 (5.00)	5.86 (2.21, 15.55)	28 (11.67)	3.06 (1.17, 7.98)	14 (5.83)	1.30 (0.44, 3.84)
3	51 (23.18)	1.76 (0.78, 3.99)	37 (16.30)	2.13 (0.99, 4.56)	33 (14.54)	2.68 (1.17, 6.16)	32 (14.10)	2.54 (1.08, 5.98)
4	—	—	17 (10.30)	1.51 (0.44, 5.21)	17 (10.30)	2.22 (0.55, 9.01)	13 (7.88)	2.02 (0.49, 8.39)
5	25 (13.97)	1.70 (0.36, 7.94)	17 (9.29)	2.66 (0.47, 15.06)	19 (10.38)	4.45 (1.31, 15.14)	16 (8.74)	4.80 (1.19, 19.26)
Adjusted for maternal age at enrollment, race, education, marital status, maternal serum cotinine concentrations at enrollment, maternal IQ, child sex, maternal depression, household income, and HOME Inventory score.								

We identified no significant statistical interactions between BDE-47 and child sex in the linear mixed models of MDI/FSIQ [*p* of the interaction term (*p*_interaction_) = 0.78], Externalizing Problem score (*p*_interaction_ = 0.17), and hyperactivity subscale score (*p*_interaction_ = 0.39). Excluding 16 mothers with BDE-47 > 100 ng/g lipid yielded results on MDI/FSIQ, Externalizing Problems, hyperactivity subscale, and Behavioral Symptoms Index similar to the full sample of 309 women (data not shown). Associations between maternal BDE-47 concentrations and measures of cognitive abilities estimated using one linear regression model for WPPSI FSIQ at age 5 years and a separate mixed model for Bayley MDI scores at 1, 2, and 3 years of age were very similar to corresponding estimates based on a single mixed model for the MDI scores and age 5 WPPSI FSIQ (see Supplemental Material, Table S5). We also tested wet-based BDE-47 and sum_4_BDEs as independent variables with adjustment for total lipids as a covariate in linear mixed models, and the results were similar to lipid-based analyses (data not shown).

## Discussion

We found evidence that prenatal exposure to brominated flame retardants, assessed from PBDE maternal serum concentrations measured at approximately 16 weeks of gestation, was associated with lower FSIQ and higher scores for hyperactive behaviors in 5-year-old children enrolled in a prospective U.S. birth cohort that had serum PBDE concentrations comparable to the U.S. average (Sjödin et al. 2008). These results confirm previous study findings that suggest that PBDEs may be developmental neurotoxicants.

The results of our study were remarkably consistent with those of two previous studies that found brominated flame retardants were associated with decrements in FSIQ and increases in hyperactive behaviors or ADHD index. The estimated decrement in FSIQ at age 5 years (–4.5; 95% CI: –8.8, –0.1) associated with a 10-fold increase in BDE-47 exposure in this study is consistent with other reports (WTC cohort: –5.5 points; 95% CI: –10.8, –0.2) at age 4 years related to a 10-fold increase in cord BDE-47 (derived from original estimate of natural log BDE-47); California farming community cohort: –4.7 points (95% CI: –9.4, 0.1) at age 7 years related to a 10-fold increase in maternal sum_4_BDEs) (Eskenazi et al. 2013; Herbstman et al. 2010). Women in the HOME study cohort had higher median prenatal BDE-47 exposure (18.9 ng/g lipid) than did the WTC cohort (11.2 ng/g lipid) (Herbstman et al. 2010) and the California farming community cohort (15.7 ng/g lipid) (Eskenazi et al. 2013). Another study in the Netherlands with much lower median prenatal BDE-47 levels (0.9 ng/g lipid) and smaller sample size (*n* = 62) did not report a correlation with WPPSI IQ at 5–6 years of age (Roze et al. 2009). We did not find an association of prenatal PBDE exposure and Bayley MDI and PDI scores in the first 3 years. Two studies, using breast milk PBDE levels, found no associations between BDE-47 and Bayley scores in infants and toddlers (Chao et al. 2011; Gascon et al. 2012). BDE-209 was not measured in most U.S. studies, including ours. Although we identified an inverse association between maternal PBDE exposure and child FSIQ at age 5 years, we did not find a significant association with FSIQ < 85.

We found that maternal PBDE exposure was related to higher risk of children having externalizing behavior problems or hyperactivity in the “at risk” range of clinical significance. This is consistent with the study from the California farming community, which found a 2–3 times higher risk of ADHD index in the “moderately or markedly atypical” range (Eskenazi et al. 2013). Two smaller studies in Europe had reported significant deficits in attention from prenatal or postnatal exposures (Gascon et al. 2011; Roze et al. 2009). Another study from North Carolina used breast milk PBDE levels (median, 28.7 ng/g lipid) and reported higher externalizing behavior domain scores and activity/impulsivity behaviors related to PBDE exposure (Hoffman et al. 2012). These results suggest that PBDEs, like lead and other environmental toxicants, may be a risk factor for the development of hyperactivity and ADHD in children (Froehlich et al. 2009; Lanphear 2012).

The potential mechanisms of PBDEs on cognitive function and hyperactivity have been postulated to include induced neuronal apoptosis, decreased neuronal migration, interrupted intracellular calcium (Ca^2+^) homeostasis, and altered neurotransmitter release and function (Dingemans et al. 2011). PBDEs and their hydroxylated metabolites (OH-PBDEs) can bind to the serum-binding proteins transthyretin and thyroxine-binding globulin, affect deiodinase enzyme activity, and alter thyroid hormone metabolism and excretion, resulting in reduced thyroxine levels in experimental animals (Butt et al. 2011; Marchesini et al. 2008; Meerts et al. 2000; Szabo et al. 2009; Zhou et al. 2002). Human studies also observed PBDE-associated thyroid hormones (thyroid-stimulating hormone, total and free thyroxine, total and free triiodothyronine) disruption during pregnancy (Chevrier et al. 2010; Herbstman et al. 2008; Lin et al. 2011; Stapleton et al. 2011; Zota et al. 2011). Thyroid disruption may be a critical underlying mechanism related to the developmental neurotoxicity of PBDEs and their metabolites (Dingemans et al. 2011); however, the recent study in the California farming community found significant associations with neurobehavioral deficits even after adjusting for maternal thyroid hormones (Eskenazi et al. 2013). This suggests that other mechanisms might also be important. In addition to thyroid receptors, PBDEs and OH-PBDEs have also been shown to bind with estrogen, androgen, progesterone, glucocorticoid, gamma aminobutyric acid A (GABA_A_), and nicotinic acetylcholine (nACh) receptors (Dingemans et al. 2011; Hamers et al. 2006; Hendriks et al. 2010).

In 2009, the United Nation Environment Programme (UNEP) added penta- and octaBDEs, two of the three existing commercial PBDE formulations, to the list of banned persistent organic pollutants (POPs) because of concerns about bioaccumulation and toxicity in wildlife and mammals (UNEP 2009). In 2004, penta- and octaBDEs were voluntarily withdrawn from the U.S. market, but products manufactured before then may still contain PBDEs, which can continue to be released into the environment by regular use, volatilization, and deposition in indoor dust. PBDEs are persistent in the environment and they bioaccumulate in the food chain. Humans are exposed to PBDEs primarily through dust ingestion and food intake (Lorber 2008; Schecter et al. 2006). U.S. women of reproductive age had GM of BDE-47 at 21 ng/g lipid in the NHANES (National Health and Nutrition Examination Survey) 2003–2004, compared with only 2–3 ng/g lipid in Europe and Asia, largely due to higher U.S. standards to prevent flammability in furniture (Kim et al. 2012; Vizcaino et al. 2011). The results from this and other observational human studies support efforts to reduce pentaBDE exposures in the U.S. population, especially for pregnant women and young children. Unfortunately, brominated flame retardants are persistent and Americans are likely exposed to higher PBDE levels than people from other parts of the world; thus, it is likely that it will take decades for the PBDE levels in the U.S. population to be reduced to current European or Asian levels.

This study has notable strengths and limitations. We successfully followed study participants from a general population with serum levels similar to U.S. adults, and examined cognitive and behavioral outcomes in a prospective birth cohort with repeated follow-ups through age 5 years (follow-up rate 95% at 1 year, 81% at 2 years, 76% at 3 years, 57% at 4 years, and 63% at 5 years of age in this study sample of 309 mother–child pairs). We were able to adjust for a variety of potential confounders, including maternal depression, maternal IQ, sociodemographic factors, and exposure to cotinine. Even so, the possibility of unmeasured confounding cannot be ruled out, especially behavioral and lifestyle factors which may affect both exposure to PBDEs and child neurodevelopment (Buttke et al. 2013). We did not have a measure for BDE-209 in maternal serum; BDE-209 is the major congener of decaBDE that is still widely used in the United States (Costa and Giordano 2011). We also did not have measures of PBDE levels in child serum, which may be higher than maternal levels (Eskenazi et al. 2013; Toms et al. 2009). Finally, we lacked a measure of OH-PBDEs, which may be more important than PBDEs because of their potency for receptor binding and structural similarity to thyroxine (Dingemans et al. 2011; Li et al. 2010; Wan et al. 2009). However, a recent report suggests a high correlation between maternal total PBDEs and total OH-PBDEs (Pearson *r* = 0.76) (Chen et al. 2013).

In summary, the results of this study confirmed those of previous studies, which showed that prenatal exposure to PBDEs is significantly associated with reduced FSIQ in children. We found that prenatal exposure to PBDEs is also significantly associated with increases in scores of externalizing problems and hyperactive behaviors in children. The research highlights the need to reduce inadvertent exposure to PBDEs in the home and office environment (e.g., via dust), and in diet (e.g., via fish or meat products), to avert potential developmental neurotoxicity in pregnant women and young children (Lorber 2008; Schecter et al. 2006). Additional research is needed to illustrate the mechanistic pathways linking PBDE exposure and neurodevelopmental deficits and to investigate BDE-209 and OH-PBDEs for human developmental toxicity. Finally, research on the neurodevelopmental toxicity of the other flame retardants used to replace PBDEs is also urgently needed.

## Supplemental Material

(286 KB) PDF
