# Supplementary material for: Prenatal Polybrominated Diphenyl Ether Exposures and Neurodevelopment in U.S. Children through 5 Years of Age: The HOME Study
Source: Environ Health Perspect. 2014 May 28;122(8):856–62. doi: 10.1289/ehp.1307562 (PMC4123029; doi:10.1289/ehp.1307562)
Supplement: (286 KB) PDF [file ehp.1307562.s001.pdf]

Supplemental Material

Prenatal Polybrominated Diphenyl Ether Exposures and Neurodevelopment in U.S. Children through 5 Years of Age: The HOME Study

Aimin Chen, Kimberly Yolton, Stephen A. Rauch, Glenys M. Webster, Richard Hornung, Andreas Sjödin, Kim N. Dietrich, and Bruce P. Lanphear

Table of Contents **Page**

Table S1. Comparisons of demographics, socioeconomic status, prenatal PBDE concentrations, and Bayley scores between 190 children followed and 119 children loss to follow-up at age 5 years. **2**

Table S2. Estimated score differences and 95% confidence intervals in Bayley Psychomotor Development Index and BASC-2 behavior rating scores at ages 1-5 years by a ten-fold increase in maternal BDE-47 concentration. **3**

Table S3. Estimated score differences and 95% confidence intervals in cognitive and behavior rating scores at ages 1-5 years by a ten-fold increase in maternal Sum_4BDEs concentration. **4**

Table S4. Estimated odds ratios and 95% confidence intervals of having cognitive and behavior rating scores one standard deviation from the population mean at ages 1-5 years by a ten-fold increase in maternal Sum_4BDEs concentration. **5**

Table S5. Estimated score differences and 95% confidence intervals of Bayley MDI and WPPSI FSIQ in separate models by a ten-fold increase in maternal PBDE concentration **6**

Table S1. Comparisons of demographics, socioeconomic status, prenatal PBDE concentrations, and Bayley scores between 190 children followed and 119 children loss to follow-up at age 5 years.

Characteristics	Followed at age 5 years	Loss to follow-up at age 5 years	p
Maternal age (years), n (%)			0.64
<25 years	39 (20.53)	24 (20.17)	
25-34 years	122 (64.21)	72 (60.50)	
≥35 years	29 (15.26)	23 (19.33)	
Maternal race/ethnicity, n (%)			0.19
Non-Hispanic White	122 (64.21)	85 (71.43)	
Non-Hispanic Black and others	68 (35.79)	34 (28.57)	
Maternal education, n (%)			0.32
High school or less	44 (23.16)	26 (21.85)	
Some college or 2-yr degree	50 (26.32)	22 (18.49)	
Bachelor's	60 (31.58)	41 (34.45)	
Graduate or professional	36 (18.95)	30 (25.21)	
Maternal marital status, n (%)			0.11
Married or living with partner	149 (78.42)	102 (85.71)	
Not married and living alone	41 (21.58)	17 (14.29)	
Household income, n (%)			0.23
<$20,000	44 (23.16)	19 (15.97)	
$20,000-79,999	96 (50.53)	61 (51.26)	
≥$80,000	50 (26.32)	39 (32.77)	
Child gender, n (%)			0.39
Male	83 (43.68)	58 (48.74)	
Female	107 (56.32)	61 (51.26)	
Maternal BDE-47, n (GM ± GSD, ng/g lipid)[a]	190 (20.52±2.58)	119 (19.45±2.67)	0.63
Maternal Sum_4BDEs , n (GM ± GSD, ng/g lipid)[a,b]	177 (37.58±2.59)	102 (37.95±2.62)	0.94
Bayley scores, n (mean ± SD)			
MDI at age 1 year	185 (93.44±9.73)	110 (94.43±10.13)	0.41
PDI at age 1 year	185 (91.51±13.33)	109 (92.56±13.25)	0.52
MDI at age 2 years	170 (90.00±14.02)	78 (89.97±12.93)	0.99
PDI at age 2 years	170 (92.36±13.44)	78 (89.50±13.52)	0.12
MDI at age 3 years	169 (93.69±13.34)	59 (92.69±14.73)	0.63
PDI at age 3 years	168 (98.00±14.50)	58 (95.86±15.81)	0.34

[a]GM: Geometric mean, GSD: Geometric standard deviation. [b]Sum_4BDEs is the sum of BDE-47, -99, -100, and -153.

Table S2. Estimated score differences and 95% confidence intervals in Bayley Psychomotor Development Index and BASC-2 behavior rating scores at ages 1-5 years by a ten-fold increase in maternal BDE-47 concentration.[a]

Assessment	β (95% CI) of log_{10}BDE-47
Bayley PDI	
1 year	0.28 (-3.49, 4.05)
2 years	2.59 (-1.67, 6.85)
3 years	0.58 (-4.37, 5.53)
BASC-2 Internalizing Problems	
2 years	-0.25 (-2.42, 1.91)
3 years	1.86 (-1.20, 4.93)
4 years	2.27 (-0.84, 5.38)
5 years	0.72 (-2.49, 3.93)
BASC-2 Adaptive Skills	
2 years	-0.14 (-2.24, 1.97)
3 years	0.43 (-1.88, 2.74)
4 years	0.56 (-2.45, 3.57)
5 years	1.49 (-1.62, 4.60)
BASC-2 Attention Problems	
2 years	-0.22 (-2.71, 2.28)
3 years	1.49 (-0.99, 3.97))
4 years	0.34 (-2.50, 3.18)
5 years	2.47 (-0.69, 5.63)
BASC-2 Aggression	
2 years	1.99 (-0.21, 4.19)
3 years	2.31 (-0.69, 5.32)
4 years	2.01 (-1.42, 5.44)
5 years	1.63 (-1.33, 4.59)
BASC-2 Anxiety	
2 years	-1.31 (-3.41, 0.78)
3 years	-0.15 (-3.43, 3.14)
4 years	2.35 (-0.83, 5.53)
5 years	-0.70 (-4.05, 2.64)
BASC-2 Withdrawal	
2 years	-0.54 (-2.91, 1.82)
3 years	-0.99 (-3.71, 1.72)
4 years	1.38 (-1.53, 4.30)
5 years	-0.29 (-3.15, 2.56)

[a]Adjusted for maternal age at enrollment, race, education, marital status, maternal serum cotinine concentrations at enrollment, maternal IQ, child sex, maternal depression, household income, and HOME Inventory.

Table S3. Estimated score differences and 95% confidence intervals in cognitive and behavior rating scores at ages 1-5 years by a ten-fold increase in maternal Sum_4BDEs concentration.[a,b]

Age	Bayley MDI/WPPSI FSIQ	BASC-2 Externalizing Problems	BASC-2 Hyperactivity subscale	BASC-2 Behavioral Symptoms Index
1 year	-0.77 (-3.84, 2.30)	--	--	--
2 years	-1.67 (-5.66, 2.31)	1.83 (-0.56, 4.22)	2.09 (-0.63, 4.80)	0.89 (-1.28, 3.06)
3 years	-1.13 (-5.14, 2.89)	2.53 (-0.32, 5.38)	3.11 (0.22, 6.00)	2.44 (-0.31, 5.20)
4 years	--	2.57 (-0.71, 5.85)	2.92 (-0.30, 6.14)	2.48 (-0.49, 5.44)
5 years	-4.38 (-8.90, 0.14)	2.70 (-0.39, 5.80)	3.35 (0.18, 6.52)	2.90 (-0.18, 5.99)

[a]Adjusted for maternal age at enrollment, race, education, marital status, maternal serum cotinine concentrations at enrollment, maternal IQ, child sex, maternal depression, household income, and HOME Inventory. [b]Sum_4BDEs is the sum of BDE-47, -99, -100, and -153.

Table S4. Estimated odds ratios and 95% confidence intervals of having cognitive and behavior rating scores one standard deviation from the population mean at ages 1-5 years by a ten-fold increase in maternal Sum_4BDEs concentration.[a,b]

Age	N (%) MDI/FSIQ <85	OR (95% CI) MDI/FSIQ <85	N (%) Externalizing Problems ≥60	OR (95% CI) Externalizing Problems ≥60	N (%) Hyperactivity subscale ≥60	OR (95% CI) Hyperactivity subscale ≥60	N (%) Behavioral Symptoms Index ≥60	OR (95% CI) Behavioral Symptoms Index ≥60
1 year	48 (18.75)	1.17 (0.47, 2.88)	--	--	--	--	--	--
2 years	71 (32.87)	0.69 (0.30, 1.57)	12 (5.56)	4.96 (1.94, 12.66)	26 (12.04)	2.90 (1.04, 8.11)	12 (5.56)	1.25 (0.41, 3.83)
3 years	49 (24.50)	1.39 (0.59, 3.31)	34 (16.50)	2.04 (0.94, 4.41)	29 (14.08)	2.28 (0.93, 5.60)	28 (13.59)	2.23 (0.88, 5.62)
4 years	--	--	16 (10.53)	2.09 (0.68, 6.44)	16 (10.52)	3.16 (0.90, 11.12)	11 (7.24)	2.24 (0.48, 10.39)
5 years	23 (13.86)	1.80 (0.39, 8.30)	17 (10.00)	3.30 (0.73, 14.83)	18 (10.59)	5.38 (1.78, 16.32)	16 (9.41)	4.40 (1.02, 18.92)

[a]Adjusted for maternal age at enrollment, race, education, marital status, maternal serum cotinine concentrations at enrollment, maternal IQ, child sex, maternal depression, household income, and HOME Inventory. [b]Sum4BDEs is the sum of BDE-47, -99, -100, and -153.

Table S5. Estimated score differences and 95% confidence intervals of Bayley MDI and WPPSI FSIQ in separate models by a ten-fold increase in maternal PBDE concentration[a]

Outcome	Log10 BDE-47	Log10 Sum_4BDEs[b]
Bayley MDI[c]		
1 year	-0.83 (-3.80, 2.14)	-0.83 (-3.90, 2.24)
2 years	-1.84 (-5.66, 1.98)	-1.84 (-5.84, 2.17)
3 years	-1.98 (-5.77, 1.81)	-1.44 (-5.49, 2.60)
WPPSI FSIQ[d]		
5 years	-4.17 (-8.91, 0.56)	-4.47 (-9.36, 0.43)

[a]Adjusted for maternal age at enrollment, race, education, marital status, maternal serum cotinine concentrations at enrollment, maternal IQ, child sex, maternal depression, household income, and HOME Inventory. [b]Sum_4BDEs is the sum of BDE-47, -99, -100, and -153. [c]Covariates adjusted Mixed model for Bayley MDI including ages 1 to 3 years. [d]Covariates adjusted linear model for WPPSI FSIQ at age 5 years.
